# Plant–microbe partnerships in 2020

**DOI:** 10.1111/1751-7915.12382

**Published:** 2016-07-15

**Authors:** Birgit Mitter, Nikolaus Pfaffenbichler, Angela Sessitsch

**Affiliations:** ^1^ Bioresources UnitHealth & Environment DepartmentAIT Austrian Institute of Technology GmbHA‐3430TullnAustria

## Abstract

The plant holobiont comprises the plant and its associated microbiota, which interact with each other and determine holobiont functioning and plant performance. We have started to understand the complexity of the involved microorganisms and their interactions, however, we need more research on plant–microbiome interactions to understand holobiont functioning. By 2020 we expect that our knowledge on these interactions will have considerably increased facilitating crop management practices based on the interactions of the plant holobiont.

Already in 1994, Richard Jefferson introduced in a symposium lecture his idea that the evolutionary selection unit is not a single organism but a macro‐organism (e.g. a plant or animal) and all its associated microorganisms that act in consortia (Jefferson, 1994). Consequently, the fitness of plants is determined by the entire suite of genes (the hologenome), consisting of the genome of the host plant as well as the genomes of all epi‐ and endophytes. Jefferson further concluded that “agriculture can only progress sustainably when balanced hologenetic combinations or holo‐alleles are present” (Jefferson, 1994).

Today, more than 20 years after Jefferson's pioneering ideas, research on the plant microbiome is blooming and the importance of the plant microbiome for plant health and development is well acknowledged (Bulgarelli *et al*., 2013; Hardoim *et al*., 2015). Those microorganisms that live in the root environment (rhizosphere and root) as well as in seeds have received most attention in this regard (Philippot *et al*., 2013; Barret *et al*., 2015; Klaedtke *et al*., 2016; Lareen *et al*., 2016). The microbial component of healthy seeds appears to be inherited between plant generations and is likely to represent an important “seed” for the microbiome build‐up, whereas soil and root‐derived microorganisms arrive later and have to compete with the already established microflora. Thus, the seed‐associated microbiota is likely to mediate different functions than root‐associated microbiota, such as effects on germination, early plant establishment and survival. In contrast to the seed microbiota, which consists of a limited range of microbial species (Truyens *et al*., 2014), the root environment hosts a tremendous microbial diversity due to the fact that root exudates and mucilage attract and select a high number of different soil microorganisms. The rhizosphere also represents the main source of microorganisms, which migrate into plants via the root and establish sub‐communities inside plants (Hardoim *et al*., 2015). Consequently, the root is a hot spot of plant–microbe interactions and the microbiome of the rhizosphere and root is especially important for plant nutrition, abiotic stress tolerance and defence against pathogen attack (Mitter *et al*., 2013; Ramírez‐Puebla *et al*., 2013). Evolution created sophisticated communication systems by which the plant influences the behaviour of microorganisms in the root environment to its own favour. For example, barley plants respond to root infection by the phytopathogenic fungi *Pythium ultimum* with increased exudation of phenolic and organic acids, which in turn induce the expression of the antifungal *phlA* in the root‐colonizing bacterium *Pseudomonas fluorescens* CHA0 (Jousset *et al*., 2011). Domestication as well as modern crop breeding together with high‐input agricultural practices have counteracted these systems and resulted in a decrease in the diversity of plant beneficial interactions and a reduction of the ability to establish such interactions in modern crop varieties (Pérez‐Jaramillo *et al*., 2016). In other words, conventional agriculture might have selected for unbalanced hologenetic combinations. For the successful re‐integration of microbial functions in agronomic management, we need to better understand the functioning of the holobiont plant and in particular the interactions of the plant and its microbial components as well as the interactions between different members of the microbial assemblage in and on the plant.

The interactions between the plant and its microbiota can be manifold and the positive effects of microorganisms on plant health and growth can be either directly, e.g. by the production of phytohormones, modulation of ethylene levels in the plant and inhibition of pathogen growth (Mitter *et al*., 2013), or indirectly, e.g. by inducing changes in the host plant gene activity (Alfano *et al*., 2007; Pinedo *et al*., 2015) or changes in microbiome composition (Ardanov *et al*., 2012, 2016). The plant itself also influences the composition and activity of its associated microbiota. The impact of the plant species, the plant developmental stage as well as the plant physiology on microbial assemblages in and on plants has been intensively studied (Mitter *et al*., 2013; Lareen *et al*., 2016) and plant genotype specificity of plant–microbe interactions has often been reported (Da *et al*., 2012; Vargas *et al*., 2012; Neiverth *et al*., 2014). More recently, researchers found out that microorganisms adjust their metabolism to adapt to the plant environment during rhizosphere or root colonization (Shidore *et al*., 2012; Alquéres *et al*., 2013; Drogue *et al*., 2014) or in response to plant stress reactions (Sheibani‐Tezerji *et al*., 2015a). All these studies point to a lively dialogue between the plant and its associated microorganisms, and in 2020 we would have obtained better understanding on the genetic determinants involved. For example, there are reports showing that inoculation with plant beneficial bacteria do not induce defence events commonly found after microbial attack in plants (Bordiec *et al*., 2011). But, how can the plants immune system discriminate between pathogenic and beneficial microorganisms? Or how can we explain that certain plant beneficial microorganisms speed up growth in many different genetically not related plant species but can show astonishing strong differences in their effect on different varieties within one plant species, i.e. *Paraburkholderia* (formerly *Burkholderia*) *phytofirmans* PsJN in potato (Da *et al*., 2012). To explore the full potential of plant–microbe partnerships for sustainable plant production, we need a better understanding of the plant genetic mechanisms that determine the plant's ability to interact with beneficial microorganisms. More systemic studies employing multidisciplinary research teams will allow us to reach this goal. By 2020, we should be able to provide molecular markers predicting beneficial plant–microbe interactions for plant breeding programmes with the aim to create the best plant–microbiome associations for optimum plant performance.

Numerous studies report on the complexity of plant microbiomes comprising multiple fungal and bacterial members (reviewed by Vorholt, 2012; Philippot *et al*., 2013; Hardoim *et al*., 2015) including pathogens and mutualists. It is obvious that such complex microbiomes are characterized by a dense network of interactions between individual members, which are still poorly understood. Few studies have provided evidence on such interactions not only between the plant host and its microbiome but also between different microorganisms playing a detrimental role on microbiome functioning. Known is the interaction between symbionts such as arbuscular mycorrhizal fungi (AMF) and rhizobia leading in combination to greater plant productivity than when applied alone (Larrimer *et al*., 2014; van der Heijden *et al*., 2016). Furthermore, representatives of *Mollicutes* and “*Candidatus* Glomeribacter” were shown to live in hyphae and spores of AMF (Bonfante and Anca, 2009; Naumann *et al*., 2010) and contribute to colonization and formation of the mycorrhizal structures in plant roots (Garbaye, 1994; Frey‐Klett *et al*., 2007). Several studies reported interactions between AMF and fungal endophytes including competition or antagonism (Eschen *et al*., 2010; Wearn *et al*., 2012), with alkaloids or allelopathic compounds produced by the fungal endophytes potentially interfering with AMF (Antunes *et al*., 2008; Mack and Rudgers, 2008). Another interesting example of multitrophic interactions is the presence of endohyphal bacteria in filamentous fungal endophytes (Hoffman and Arnold, 2010), resulting in altered functioning. For example, the endohyphal bacterium *Luteibacter* sp. was found to greatly enhance indole‐3‐acetic acid (IAA) production of the fungal endophyte, although the bacterium does not show IAA production when grown alone (Hoffman and Arnold, 2008). Well known is also the interaction between the phytopathogen *Rhizopus microsporus*, the causal agent of rice seedling blight, and its interaction with the endofungal bacterium *Burkholderia endofungorum*, which is responsible for toxin production (Partida‐Martinez and Hertweck, 2005). It furthermore has been demonstrated that viruses interact with plant‐associated microbiota. The geothermal grass *Dichanthelium lanuginosum* as well as its fungal endophyte *Curvularia protuberata* can tolerate temperatures of 40°C when grown separately, but in symbiosis, the combination of the host plant and the fungal endophyte infected with a virus can tolerate soil temperatures as high as 65°C (Márquez *et al*., 2007; Rodriguez *et al*., 2008).

These examples demonstrate how important it will be to shed more light on the interplay between microbiome members with regard to interacting with the plant, and a multitude of yet unknown interactions impacting plant growth and health are still to be expected. A better understanding on the type of interactions, the mechanisms and signalling cascades involved will be applicable in different directions. Defined microbial consortia comprising bacteria as well as fungi may be designed to provide a specific set of functions. Understanding the interactions between pathogens or pests with other plant‐associated microbiota may lead to novel avenues in plant disease prevention (Massart *et al*., 2015), which are urgently needed to reduce pesticide input and to overcome the development of resistance.

By 2020, we should have acquired better knowledge on the ecology and functioning of plant–microbiome interactions in different plant compartments. Of main interest are the root environment and reproductive organs such as seeds (Fig. 1). Our understanding on which microbial traits are needed to preferentially colonize seeds, roots or other plant tissues is still limited. One bottleneck is that (cultivation‐independent) microbiome analysis is mostly based on the analysis of 16S rRNA genes, which provides information at the species or genus level. However, various important characteristics with regard to interaction with the plant such as plant tissue or plant genotype preferences, mutualism or competitive ability are manifested at the strain rather than at the species level. This becomes evident when investigating different isolated strains belonging to the same species (Idris *et al*., 2004; Sheibani‐Tezerji *et al*., 2015b). Therefore, it will be important that in 2020, we will have strain‐dependent tools available to monitor microbiome members, and to have a better understanding on the determinants for establishment in a particular tissue. Current sequencing efforts, genome comparison, improved tools in genome annotation, combined with classical microbial genetics and detailed functional analysis will lead to advanced knowledge on which microorganisms will perform particular plant growth‐promoting functions and will thrive/compete in a particular environment. From an application point of view, this information will lead to tailored microbial applications for crop enhancement, which will improve the reliability and field success of such applications. Depending on the target microbial trait (e.g. improvement of seed germination or antagonism of soil‐borne pathogens), it will be possible to select appropriate strains showing these functions with the desired plant genotype(s), to establish well in the target environment (e.g. in/on seeds for seed germination, in the root environment for pathogen antagonism), and to also express the target traits in the relevant environment.

**Figure 1:**
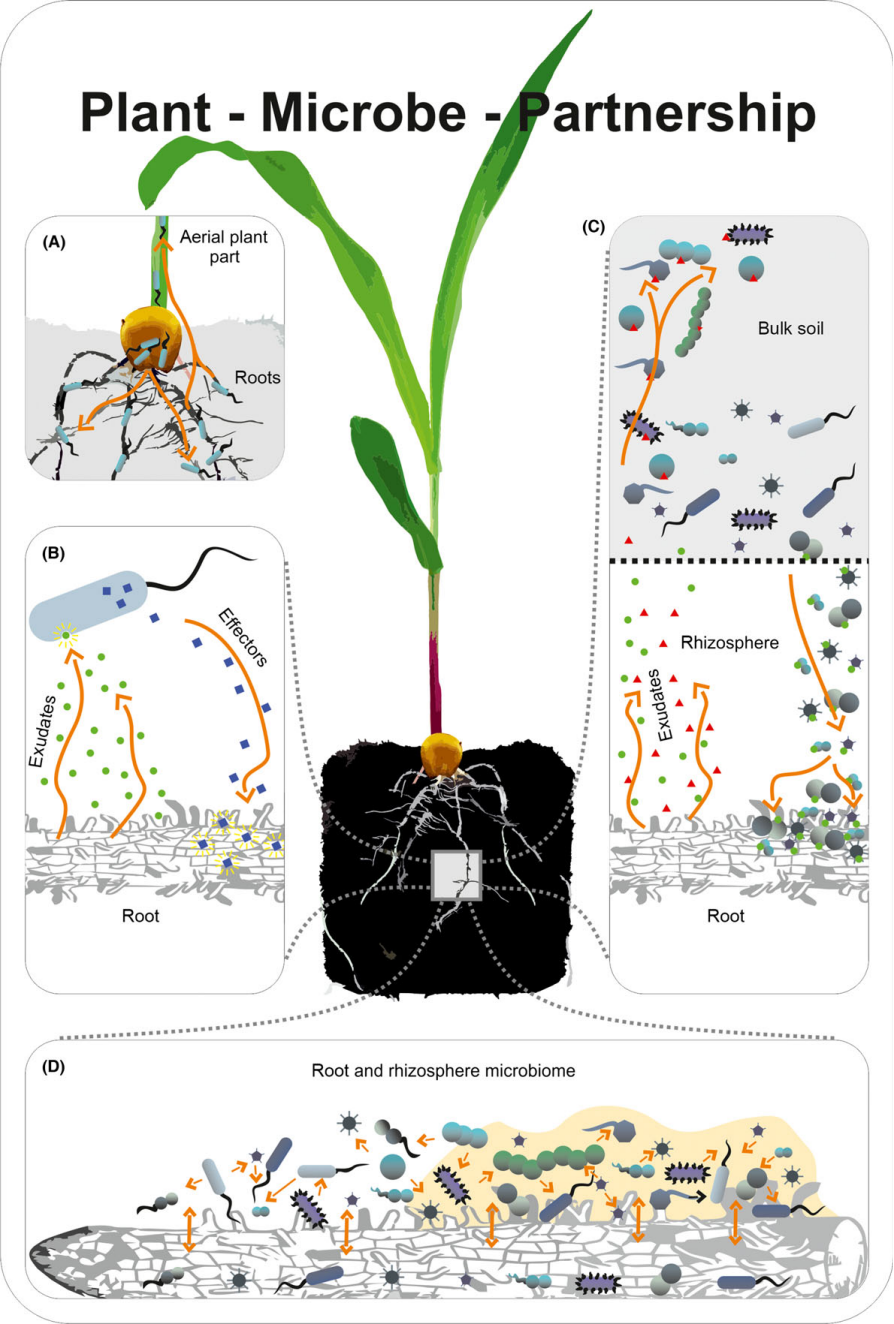
Close‐up view of plant–microbe partnership. (A) Plants are colonized initially by microbes originating from the seed. This seed‐derived microbiota is complemented and partly substituted gradually by rhizosphere microorganisms migrating into the plant via roots. Plant–microbe partnerships occur at different levels of complexity. (B) The plant interacts with single organisms. It responds to the presence of a microbe and its metabolites and *vice versa* the microbe is affected by the plant environment and reacts to plant metabolism and physiology. (C) The plant interacts with the microbiota in the soil and rhizosphere. Plant exudates attract microbes in the soil thereby directing a subset of them to the root zone. In turn, the activity of the microbiota in the root zone has strong impact on plant growth and health. (D) The microorganisms within the root and rhizosphere microbiota dynamically interact with each other and the microbiota in the root.

Our understanding of higher organisms is changing fundamentally. Plants are no longer understood as monogenetic individuals but as polygenetic entities, in which the microbiota play a central role in fitness, adaption and diversification of the holobiont (Bordenstein and Theis, 2015). By 2020, we should have developed a better understanding of the functioning of the holobiont plant on the basis of fundamental knowledge on plant–microbiome interactions. This will allow realizing Jefferson's vision of a sustainable agriculture based on balanced hologenetic combinations in future.

## Conflict of interest

Authors have no conflict of interest to declare.
